# Community based countrywide analysis of lactase persistence related genetic variants and their correlation with digestive symptoms in Libya

**DOI:** 10.1371/journal.pgph.0006386

**Published:** 2026-05-04

**Authors:** Ariej M. Mustafa, Inas M. Alhudiri, Abdenaser A. Mohamed, Hafsa Al-Emam, Ahmad Ramadan, Halla Elshwekh, Hamza Ben Zeglam, Nabil S. Enattah

**Affiliations:** 1 Department of Genetic Engineering, Libyan Biotechnology Research Center, Tripoli, Libya; 2 Department of Environment, Food and Water Sciences, Libyan Biotechnology Research Center, Tripoli, Libya; UMass Chan Medical School - Baystate Medical Center, UNITED STATES OF AMERICA

## Abstract

Lactose intolerance is a complex symptomatic response to lactose maldigestion that is influenced by, but not strictly determined by, the down- regulation of the enzyme lactase. Several nucleotide polymorphisms *− 13910C > T (rs4988235), − 13907C > G (rs41525747), − 13915T > G (rs41380347), − 14010G > C (rs145946881)* ***and −14009T > G*** *(rs869051967)* upstream of the LCT gene have been associated with lactase persistence in European, African and Middle Eastern populations. We aimed to study the prevalence of lactase-persistence associated variants of the LCT gene in the Libyan population and its correlation with digestive symptoms (heartburn, diarrhea, nausea, bloating, abdominal cramps,).Buccal DNA swabs were collected from 242 adult individuals from the western, southern and eastern regions of Libya. A 500 bp DNA region spanning the *− 13910C > T* variant was analyzed. Novel variants (-13883T > A, -13921A > C, -13961T > C and -13962C > A) were detected in the analyzed region in addition to the previously described *− 13910C > T* and *−13915T > G* variants. However, their functional impact on lactase expression remains to be determined. The prevalence of the lactase persistence phenotype was associated with the derived alleles −13910*T and −13915*G, which were collectively detected in 30% of the study participants. The allele frequency of the lactase persistence -13915*G variant was 0.133 (SD ± 0.016), whereas the frequency of the -13910*T allele was 0.029 (SD ± 0.008). The most common was the Arab variant *− 13915T > G* in the eastern region of Libya followed by the European *− 13910C > T* variant. Both novel and well-known variants of LCT gene associated with lactase persistence are common in Libyans. Further studies are needed to confirm the association of the novel discovered variants with lactase persistence.

## Introduction

Adult-type hypolactasia, or lactase non-persistence (LNP), is the ancestral mammalian phenotype characterized by the post-weaning downregulation of lactase activity to approximately 10% of neonatal levels [1–3]. The age of LP downregulation is highly variable, observed from the ages of 1 week to 13 years [4]. Clinical symptoms include diarrhea, bloating, and flatulence, with prevalence rates varying significantly by ethnicity—being lowest in Northern Europeans and highest in Asian and African populations [5–8].

The lactose in mammalian milk is hydrolysed to glucose and galactose by the enzyme lactase-phlorizin hydrolase (LPH). LPH is a key glycoprotein expressed at the brush border membrane of epithelial cells lining the small intestinal wall [9].

LPH is synthesized as a large precursor that undergoes complex proteolytic processing and glycosylation to become a mature enzyme [1]. The mature enzyme has two activities: hydrolysing lactose to glucose and galactose, and hydrolysing aryl and alkyl β-glycosides to phlorizin and β-glycosylceramides [2,10] Beyond genetics, recent evidence suggests that the gut microbiome may modulate the severity of these symptoms in LNP individuals [11,12].

The lactase gene encoding the LPH enzyme has been mapped to the long arm of chromosome 2 [13]. Two SNPs, *− 13910C > T* (rs4988235) and -22018G > A (rs182549), located 14 kb and 22 kb upstream from the lactase (LCT) gene initiation codon, respectively, have been found to be co-segregated completely with the adult-type hypolactasia trait in Finnish families [6,14–17]*.* The -13910*T variant located in the enhancer region of the lactase gene (LCT) is largely associated with lactase persistence (LP) in Eurasian populations, but it is nearly absent in Sub-Saharan African populations, among whom there is a high prevalence of LP [18]. Other variants correlated with LP were later identified near the *− 13910C > T (rs4988235)* variant, namely*, − 13907C > G (rs41525747), − 13915T > G (rs41380347), − 14010G > C (rs145946881)* *and −14009T > G* *(rs869051967)* in Middle Eastern and East African populations [19,20].

The regulation of lactase activity has been studied using human and rat LCT promoter-reporter gene constructs in Caco-2 cells. DNA fragments from the SNP region were characterized with respect to their ability to regulate promoter activity when cloned upstream of the 2-kb fragment of the rat lactase promoter and assayed in intestinal Caco-2 cells. In such cells, the -13910*T variant was found to be four-fold more powerful in enhancing the LPH promoter than the -13910C variant [21].

The hydrogen breath test is widely used for diagnosis of hypolactasia, but it has some limitations, including variable sensitivity (69–100%) and specificity (89–100%) [10]. Moreover, there is a high false-negative rate of 11–30% [22]. Recently, guidelines have been established for compliance with standard criteria for hydrogen breath testing after lactose ingestion, including an increase of ≥20 parts per million of hydrogen over the baseline level as a diagnostic threshold [23]. Optimal sampling intervals, simplified protocols [24], dietary restrictions and fasting, appropriate lactose dosages for adults and children, and exclusion of antibiotics or intestinal infections were also recommended [23,24]. Using SNP genetic testing of patients with suspected LNP may therefore aid in confirming the diagnosis.

To determine the type and prevalence of genetic variants among Libyans, we sequenced the 500-bp region covering the previously identified *− 13910C > T* variant and correlated the genotypes to clinical symptoms related to ingestion of dairy products.

## Materials and methods

### Ethics statement

The study was approved by the local ethics committee of the Libyan Biotechnology Research Center, Tripoli (Ref No; NBC: 001.H.23.2), and written informed consent was obtained from all the participants or their parents/guardians for participants younger than 18 years. Subsequently, data collection took place from (25 January 2016–18 March 2016).

### Study population

The study was done on 242 adult Libyan volunteers recruited using a convenience sampling approach from a total of 32 different cities/towns in the eastern and western regions, of whom 93 were males (39.9) and 149 females (61.6%) Of the 242 recruited volunteers, 9 were excluded because they did not record their tribal origin. Therefore, the study was carried out on 233 volunteers.

Due to poor security situation, we were unable to recruit participants from the southern region, However, our focus was on the geographic region of origin rather than current residence; therefore, we included individuals currently living in East and West regions whose origin are from the southern region.

The volunteers were recruited at supermarkets, hospitals, clinics, universities and companies.

A self-reported questionnaire was used to record age, sex, tribal origin, place of residence, presence of abdominal symptoms, and milk drinking habits, Weight and height were measured, and buccal swabs were obtained from all the recruited volunteers.

### Sample size and statistical power calculation

This study was designed to estimate population allele frequencies of lactase-persistence variants. The overall study sample size was determined prior to recruitment using approximate allele-frequency estimates from other Arab countries (Morocco, Jordan, Egypt, and Saudi Arabia) shown in S2 Table for the G₁₃₉₁₅ and T₁₃₉₁₀ variants. Sample size was calculated using the formula n = z²·p(1 − p)/m² (z = 1.96), Using a conservative allele frequency of p = 0.13 for -13910*T in Libya, a sample of 174 participants is required to achieve ±5% precision. For G₁₃₉₁₅, with a conservative allele frequency of p = 0.08, a sample of 114 participants is required for ±5% precision.

Additionally, post-hoc analysis precision targets were specified using n = z²·p(1 − p)/m² (z = 1.96), where m is the desired 95% CI half-width in absolute percentage points. Using our observed frequencies (-13915*G p = 0.133; -13910*T p = 0.029), ± 10 pp precision would require approximately 45 and 11 participants, respectively; ± 5 pp would require approximately 178 and 44.

### Genotype determination

Genomic DNA was extracted from buccal swabs using Isohelix Buccal DNA Isolation Kit (Cell Projects) according to the manufacturer’s instructions and it was checked by agarose gel electrophoresis. Excess salts, primers, and dNTPs were removed by using QIAquick Purification Kit (Qiagen) according to the manufacturer’s protocol before PCR amplification.

The 500-bp region of intron 13 of the MCM6 gene spanning the *− 13910C > T* variant was amplified by PCR using forward primer 5’ GTC ACT TTG ATATGA TGA GAG CA 3’ and reverse primer 5’ CCT CGT TAA TAC CCA CTG ACC TA 3’ [19]. Each 50-µl PCR reaction premix contained 10 µl of 5X GoTaq reaction buffer, 1 µg of 1.5 mM MgCl_2_, 1 µl of 200 µM/ml dNTPs, 1.25 µl of 250 µM/ml of each primer, and 0.25 µl (0.8 U) of GoTaq DNA polymerase. DNA template (2 µl) was added before placing the tube in an Applied Biosystems GeneAmp PCR System 9700. A denaturation pre-cycle at 95°C for 2 min was followed by 34 cycles of denaturation at 95°C for 35 s, annealing at 53°C for 35 s, and extension at 72°C for 35 s. The program was terminated with a final extension at 72°C for 7 min, and then the temperature was dropped to 4°C. The PCR product was checked by agarose gel electrophoresis.

The purified PCR products were sequenced using BigDye Terminator v3.1 Cycle Sequencing kit (Applied Biosystems) according to manufacturer’s instructions. Cycle sequencing started with a denaturation pre-cycle at 96°C for 1 min followed by 25 cycles of denaturation at 96°C for 10 s, annealing at 55°C for 5 s, and extension at 60°C for 4 min. The products were purified using BigDye XTerminatorPurification kit (Applied Biosystems) followed by capillary electrophoresis in a Genetic Analyzer 3500xL (Applied Biosystems) and base calling using Sequencing Analysis Software v5.1. Sequencing traces were imported in Sequencher (Gene Codes, Ann Arbor, MI). Traces were then aligned and compared with the reference sequence. SNPs were identified, examined and recorded [25]. Nucleotide sequences for samples 132,140,147 and 207 were submitted to GenBank under the following accession numbers respectively, PP236378, PP236379, PP236380 and PP236381.

### Statistical analysis

All statistical analyses were performed using the SPSS for Windows, Version 20 (SPSS Inc., Chicago, and Illinoi, USA). Frequency differences and departure from Hardy-Weinberg equilibrium were analyzed with Pearson#39;s Χ² test. A p-value < 0.05 was considered to be statistically significant for all analyses. Accordingly, the frequencies of *−13910C > T* and *−13915T > G* polymorphisms were tested by Pearson#39;s Χ² test, which was also used to quantify the association between lactose intolerance and these polymorphisms. In addition, association between age, weight, height, body mass index (BMI), gender, origin, location, milk drinking, duration of abdominal symptoms, smoking status, diabetes, were quantified using Pearson#39;s x^2^ test.

## Results

The study was carried out on 233 volunteers. Mean age was 34.1 years ± 1.29 SD in males and 32.3 years ± 1.1 SD in females. The highest percentage of participants (43%) was found in the age group 15–30 years.

The geographic locations of tribal origin of the volunteers were as follows: 127 from the eastern region (54.5%), 81 from the western region (34.7%), and 25 from the southern region (10.7%). The majority of subjects participated in the study were located in Tripoli (85 subjects).

Of the 220 DNA samples that were successfully sequenced, 12 (5.5%) showed the heterozygous *− 13910C > T* genotype indicating LP. In which ‘C’ represents the ancestral allele associated with LNP, while ‘T’ is the derived variant associated with LP.The prevalence of this LP-associated variant was 6.7% in the western region, 3.8% in the eastern region, and 4.5% in southern region. There was no statistically significant association (p = 0.66) between the prevalence of the *− 13910C > T* genotype and region of origin using Fisher’s exact test (Table 1). No significant deviation from Hardy-Weinberg equilibrium (HWE) distribution was detected (p = 0.680).

**Table 1:** Prevalence of the normal and the *− 13910C > T* variant genotypes according to the region of origin.

Region	CC	CT	TT	Total n	p value
Western	111 (93.3%)	8 (6.7%)	0	119	0.661
Southern	21 (95.5%)	1 (5.5%)	0	22	
Eastern	76 (96.2%)	3 (3.8%)	0	79	
Total	208 (94.5%)	12 (5.5%)	0	220	

On the other hand, the prevalence was high for the *− 13915T > G* variant (21.3%) and was 2.3% for the *− 13915G > G* variant (Table 2). Of the 219 samples successfully sequenced, 52 showed the heterozygous − 13915T > G and homozygous − 13915G > G genotypes indicate lactase persistence (LP), with an overall prevalence of 24.5%. The prevalence of the *− 13915T > G* variant was highest (38.8%) among those originating from the eastern region, to 13.6% in South, and there was a statistically significant association between the *− 13915T > G* genotype and the regions of origin (p = 0.003). No significant deviation from Hardy-Weinberg equilibrium distribution (HWE) was detected (p = 0.44).

**Table 2:** The percent prevalence of the lactase-persistence associated *− 13915T > G* genotype within the three regions of origin.

Origin	TT	TG	GG	Total n	p value
West	99 (83.2%)	20 (16.8%)	0 (0.0%)	119 (100%)	0.003
South	19 (86.4%)	2 (9.1%)	1 (4.5%)	22 (100%)	
East	49 (61.3%)	25 (33.8%)	4 (5.0%)	78 (100%)	
Total	167 (76.5%)	47 (21.3%)	5 (2.3%)	219 (100%)	

An exploratory analysis suggested a potential association between BMI and the -13910*T variant (p = 0.03) Table 3. However, this finding should be interpreted with caution due to the limited number of individuals (n = 7) carrying this genotype, which results in low statistical power. Further studies with larger sample sizes are necessary to validate our observation. The CC genotype associated with lactase non-persistence tend to be associated with a normal BMI. The *− 13915T > G* genotype on the other hand was not significantly associated with BMI in any of the regions studied (p > 0.05).

**Table 3:** The relationship between *−13910C > T* and body mass index Genotype *− 13910C > T.*

Origin		Body mass index					Total	p value
		> 18-25	> 25-30	> 30-35	>35-40	> 40		
West	CC	39	38	13	1	2	93	0.03
	CT	2	1	3	1	0	7	
	Total	41	39	16	2	2	100	
South	CC	6	7	3	1		17	0.724
	CT	0	1	0	0		1	
	Total	6	8	3	1		18	
East	CC	15	20	9	1	2	47	0.859
	CT	0	1	0	0	0	1	
	Total	15	21	9	1	2	48	

During this study, four novel polymorphisms were discovered within a 79-bp region near the LP-associated variants (*−13910C > T* and *−13915T > G*): T/A-13883, T/C-13961 and C/A-13962 were detected once, and A/C-13921 was detected twice. Notably, all of them were in individuals from the eastern region. These variants were not found in the SNP database [26]. However, they remain of undetermined functional significance and unknown phenotypic relevance to LP.

Variant details are shown in S1 Table.

Based on our analysis of the associated variants *− 13910C > T* and *−13915T > G*, the prevalence of the LP associated genotypes in the Libyan population is estimated at about 30%. The allele frequency of the LP variant G-13915 in the Libyan population was 0.133 ± 0.016 SD and that of the T-13910 LP allele 0.029 ± 0.008 SD. The novel variants detected once in this study had an allele frequency of 0.0023 ± 0.0027 SD and the one detected twice had a frequency of 0.0045 ± 0.0036 SD.

### Association of *−13915T > G* (rs#41380347) with other symptoms

The most frequent LP-associated variant observed in this study, the −13915*G, was associated with the presence of abdominal symptoms (p = 0.005). Interestingly, exploring the relationship of −13915*G variant with some conditions revealed some region-related associations. First, a significant statistical association was detected in the western region between the −13915*G allele and a family history of celiac disease, where this specific variantseemed to be protective (OR = 0.828; 95% CI 0.762-0.899). This suggests that the presence of the LP-associated allele -13915*G may be linked to a reduced report of celiac disease in this subgroup, although this remains an exploratory finding. Second, in the eastern region we found a significant association between the *− 13915T > G* frequency and a family history of suffering from stomach symptoms when consuming milk (p = 0.02), which is in agreement with the results of having abdominal symptoms when consuming milk or its products or other types of food.

We found no significant association between *−13915T > G* and milk drinking habits, duration of the symptoms, family history of helicobacter, family history of diabetes, or smoking history.

## Discussion

We assessed the frequency of the LP associated genotypes based on variants of the LCT gene. The overall frequency of -13910*T and -13915*G was 30%. Similar results have been reported from many countries, such as Finland, Saudi Arabia, Morocco, Sudan, Egypt, Yemen and Oman [27–29]. Our results show that the Arab variant *− 13915T > G* is the most common LP-associated variant in the Libyan population, especially in the eastern region. **This could reflect the influence of the migration of Arab tribes** [30]. The European variant T-13910, was found with low frequency, which could reflect a small contribution of the Europeans to the Libyan genetic pool.

We detected four novel variants near *−13910C > T* and *−13915T > G*, all of them were in individuals from the eastern region. These SNPs were *−* 13883T > A, *−* 13961T > C and *−*13962C > A (detected once) and *−*13921A > C (detected twice). These SNPs were not found in the Global Lactase Persistence Association Database. Although these variants are located within the known enhancer region upstream of LCT, their functional significance remains unknown.

Interestingly, one individual had a combination of *−13883T > A,* *− 13915G > G* and *−13962C > A*. We found that her genotype is associated with LP, but her clinical history revealed that she has never drank milk as an adult, Although she reported to had digestive symptoms similar to those caused by lactose intolerance. These symptoms are most likely due to other underlying gastrointestinal factors. But these are unrelated to lactose. Also, two individuals from the eastern region had a combination of *−13910C > T* and *−13915T > G* heterozygous genotypes with symptoms of lactose intolerance. These results might indicate that the presence of more than one variant within the enhancer region may interfere with the expression of the lactase enzyme and result in lactase -non-persistence. The more likely explanation is that in the heterozygote state, the lower levels of lactase production are sufficient to induce symptoms of lactose intolerance in some individuals. Functional studies have not suggested that compound heterozygotes are compromised due to interactions. So, detailed study of the enhancer region of the LCT gene is needed to assess the effect of variants on the lactase persistence phenotype.

Since LP individuals are not on a restricted diet compared to LNP individuals, who are more careful with their diet, we analyzed the relation between BMI and LP variants. In the western region, we found a significant association between higher BMI and the 13910*T variant. Our data are in agreement with several previous studies that provide evidence for association between genetically defined LP and BMI in four European populations. These studies show that the C/C-13910 LNP genotype is associated with decreased BMI compared with *−13910C > T* / *− 13910T > T* [31–34].

On the other hand, the Arab variant *− 13915T > G* in our study was not significantly associated with BMI in any of the three regions. This result might be in agreement with a Danish study showing that high milk intake is not genetically associated with overweight/obesity *via* LP [35].

The frequency of the European T-13910 allele in Libya ranged from 2.3% ± 2.0% SD in the southern region to 3.4% ± 1% SD in the western region. S2 Table compares the frequencies of the T-13910 and G-13915 alleles in Libyans with other Arab populations. In general, the European T-13910 allele frequency in Arab populations is < 10%, except in Fulani Sudanese who have the highest frequency (48% ± 5%), followed by Saharawi (26% ± 4% SD), and Moroccan (17% ± 3% SD) [19,30]. On the other hand, the Arab G-13915 allele frequency in Libya ranged from 9% ± 4% SD to 21% ± 3% SD, which resembles the frequencies in Palestinians, Iraqis, Syrians, Lebanese, Sudanese, and Arabs of northern Oman [19,29,36–38].

This study has several limitations. First, functional assessment of lactose intolerance using hydrogen breath testing was not performed due to logistical constraints. Therefore; our study focused solely on genetic determinants of lactase persistence. However, it is increasingly recognized that the LNP phenotype is not determined by genetics alone; epigenetic regulation, such as DNA methylation of the *LCT* promoter, may influence the timing and degree of lactase downregulation [39]. Furthermore, the lack of functional testing means we could not account for the role of the gut microbiome, which can significantly mitigate clinical symptoms in LNP individuals through adaptive fermentation [11,12]. Second, although we aimed to represent all geographic regions of Libya, recruitment from the southern region was limited by security concerns. To address this, we included individuals currently residing in East and West regions whose ancestral origins are from the southern region. This is particularly relevant given the high diversity of LP variants across the Saharan and Arabian regions [7,8] Third, recruitment was convenience based sampling which may limit the generalizability of the findings to the entire Libyan population, especially as city-level sample sizes were small, so allele-frequency estimates are presented descriptively without formal statistical comparisons. Finally, while the overall study sample size was determined prior to recruitment, precision and power calculations were not performed separately for individual variants; post-hoc analyses using the observed allele frequencies indicate that the total sample of 242 participants provides sufficient accuracy for estimating pooled allele frequencies. Therefore, and hence further larger studies should be conducted to also investigate the relationship between LCT variants and metabolic markers like BMI in the Libyan population. Despite these limitations, the study provides valuable insights into the distribution of lactase-persistence variants in the Libyan population.

To the best of our knowledge, this is the first study in Libya to determine the prevalence of LP variants and their correlation with digestive symptoms. Our study revealed the presence of four novel variants (T/A-13883, A/C-13921, T/C-13961, C/A-13962) in individuals from the eastern region of Libya. **Notably, these variants were** not reported in the Global Lactase Persistence Association Database, and not found in gnomAD and 1000 Genomes

The overall frequency of the LP-associated variants *− 13910C > T* and *−13915T > G* in our sample population of different origins was 30%. The most common LP variant was the Arab variant *− 13915T > G* in the three Libyan regions, followed by the European *− 13910C > T* variant.

## Supporting information

S1 Table Variant details [40–44]. The table shows the details of the identified genetic variants, including the novel variants discovered in this study which were not found in the SNP database. The table columns show: the reference SNP ID, HGVS (GRCh38.p14), mRNA change and location, clinical significance (ClinVar), and protein changes. All HGVS nomenclatures were verified using Mutalyzer (github.com/mutalyzer/mutalyzer/wiki/Mutalyzer_explain.pdf). ^Gene Details: MCM6 (minichromosome maintenance complex component 6) gene, Location: Chromosome 22q13.1.
(DOCX)

S2 Table The allele frequencies in Libya and other Arab countries. The table shows the allele frequencies for different region in Libya (East, West, and South) as well as other Arab countries (Morocco, Jordan, Egypt, and Saudi Arabia). The table columns show: Libyan region and Arab countries and populations, n = Number of chromosomes analyzed, Allele frequencies (Percentage and standard deviation), and the reference number used for the table details.
(DOCX)
