# Supplementary material for: Community based countrywide analysis of lactase persistence related genetic variants and their correlation with digestive symptoms in Libya
Source: PLOS Glob Public Health. 2026 May 4;6(5):e0006386. doi: 10.1371/journal.pgph.0006386 (PMC13138664; doi:10.1371/journal.pgph.0006386)
Supplement: S1 Table — The table shows the details of the identified genetic variants, including the novel variants discovered in this study which were not found in the SNP database. The table columns show: the reference SNP ID, HGVS (GRCh38.p14), mRNA change and location, clinical significance (ClinVar), and protein changes. All HGVS nomenclatures were verified using Mutalyzer (github.com/mutalyzer/mutalyzer/wiki/Mutalyzer_explain.pdf). ^Gene Details: MCM6 (minichromosome maintenance complex component 6) gene, Location: Chromosome 22q13.1. (DOCX) [file pgph.0006386.s001.docx]

**S1 Table. Variant details**

| Sample ID | rs# |  | HGVS (GRCh38.p14) * | mRNA change and location | Clinical Significance (ClinVar) | Protein change |
| --- | --- | --- | --- | --- | --- | --- |
| 147 | rs1679773081 | C/A-13962 | NC_000002.12:g.135851128G>T | NM_005915.6:c.1917+274C>A | Not Reported in ClinVar | MCM6^: Intron Variant, Unregistered Allele |
| 207 | rs1679773035 | T/C-13961 | NC_000002.12:g.135851127A>G | NM_005915.6:c.1917+275T>C | Not Reported in ClinVar | MCM6 : Intron Variant |
| 140 | Novel | A/C-13921 | NC_000002.12:g.135851087T>A | NM_005915.6:c.1917+315A>T | Not Reported in ClinVar | MCM6: Intron Variant, Unregistered variation |
| 132 | Novel | A/C-13921 | NC_000002.12:g.135851087T>A | NM_005915.6:c.1917+315A>T | Not Reported in ClinVar | MCM6: Intron Variant, Unregistered variation |
| 147 | rs41380347 | T/G-13915 | NC_000002.12:g.135851081A>C | NM_005915.6:c.1917+321T>G | VCV000007688.2 -  Interpretation: association​ - but no assertion criteria | MCM6 : Intron Variant \| Enattah NS et al 2008 - PMID: 18179885 |
| 207 | rs4988235 | C/T-13910 | NC_000002.12:g.135851076G>T | NM_005915.6:c.1917+326C>A | VCV000007685.6 -  Interpretation: association​ - but no assertion criteria | MCM6 : Intron Variant \| Enattah NS et al 2002 - PMID: 11788828 |
| 147 | Novel | T/A-13883 | NC_000002.12:g.135851049A>T | NM_005915.6:c.1917+353T>A | Not Reported in ClinVar | MCM6: Intron Variant, Unregistered variation |

* All HGVS nomenclatures were verified using Mutalyzer (github.com/mutalyzer/mutalyzer/wiki/Mutalyzer_explain.pdf).

^Gene Details: MCM6 (minichromosome maintenance complex component 6) gene, Location: Chromosome 22q13.1
