# Supplementary material for: Community based countrywide analysis of lactase persistence related genetic variants and their correlation with digestive symptoms in Libya
Source: PLOS Glob Public Health. 2026 May 4;6(5):e0006386. doi: 10.1371/journal.pgph.0006386 (PMC13138664; doi:10.1371/journal.pgph.0006386)
Supplement: S2 Table — The table shows the allele frequencies for different region in Libya (East, West, and South) as well as other Arab countries (Morocco, Jordan, Egypt, and Saudi Arabia). The table columns show: Libyan region and Arab countries and populations, n = Number of chromosomes analyzed, Allele frequencies (Percentage and standard deviation), and the reference number used for the table details. (DOCX) [file pgph.0006386.s002.docx]

**S2 Table:** The allele frequencies in Libya and other Arab countries

| **Population** | **n^a^** | **Allele frequencies % (± SD)** | | **References** |
| --- | --- | --- | --- | --- |
|  |  | **T_-13910_** | **G_-13915_** |  |
| **Libya** | **440** | **0.029 (0.008)** | **0.133 (0.02)** | Current study |
| **East** | 158 | 0.025 (0.01) | 0.21 (0.03) | Current study |
| **West** | 238 | 0.034 (0.01) | 0.08 (0.02) | Current study |
| **South** | 44 | 0.023 (0.02) | 0.09 (0.04) | Current study |
| **Saudi Arabia** | **248** | **0.004 (0.004)** | **0.570 (0.03)** |  |
| **Region 1** – central | 50 | - | ***0.56 (0.07)*** | ***41*** |
| **Region 2 -** South | 50 | - | ***0.50 (0.07)*** | ***41*** |
| **Region 3**-west | 50 | - | ***0.68 (0.07)*** | ***41*** |
| **Region 4** –East | 48 | - | ***0.60 (0.07)*** | ***41*** |
| **Region 5**- North | 50 | - | ***0.48 (0.07)*** | ***41*** |
| **Jordan** | 112 | 0.054 (0.02) | 0.054 (0.02) | ***41*** |
| **Morocco** | 24 | 0.21 (0.08) | 0.083 (0.06) | ***41*** |
| **Mahas Sudan** | 30 | 0.00 (0.000) | 0.17 (0.07) | ***41*** |
| **Arabs ^b^** | 40 | 0.13 (0.05) | 0.105 (0.05) | ***41*** |
| **Arabs ^b^** | 102 | 0.098 (0.03) | ND | ***41*** |
| **Fulani (Sudan)** | 88 | ***0.48 (0.05)*** | ND | ***41*** |
| **Morocco** | 180 | 0.173 (0.03) | ND | ***41*** |
| **Saharawi** | 114 | 0.263 (0.04) | ND | ***41*** |
| **Yemen** | **496** | **0.016 (0.01)** | **0.542 (0.02)** |  |
| **Yemen 1 (Al-Akhkum)** | 86 | - | ***0.651 (0.05)*** | ***42*** |
| **Yemen 2 (Hudeida )** | 132 | - | ***0.591 (0.04)*** | ***42*** |
| **Yemen 3 (Hajja )** | 68 | - | ***0.618 (0.06)*** | ***42*** |
| **Yemen4 (Wadi Hadramawt )** | 80 | 0.038 (0.02) | 0.263 (0.05) | ***42*** |
| **Yemen 5 (Soqotra )** | 130 | 0.046 (0.02) | ***0.585 (0.04)*** | ***42*** |
| **Arabs Rashaayda (Sudan)** | 104 | - | ***0.769 (0.04)*** | ***42*** |
| **Arabs Sudan** | 92 | - | 0.076 (0.03) | ***42*** |
| **Arabs Baggara (Tchad)** | 54 | 0.019 (0.019) | ***0.463 (0.07)*** | ***42*** |
| **Arabs Shuwa (Nigeria)** | 106 | 0.085 (0.03) | 0.066 (0.02) | ***42*** |
| **Arabs Egypt** | 68 | - | 0.015 (0.01) | ***42*** |
| **Shagia Sudan** | 128 | 0.023 (0.01) | - | ***42*** |
| **Ajman Kuwait** | 74 | 0.01 (0.013) | ***0.57 (0.058)*** | ***40*** |
| **Mutran Kuwait** | 58 | 0.000 (0.000) | ***0.55 (0.065)*** | ***40*** |
| **Arabs of Northern Oman** | 684 | 0.013 (0.004) | 0.136 (0.013) | ***44*** |
| **Omanis of Asian origin** | 192 | 0.156 (0.026) | 0.000 (0.000) | ***44*** |
| **Dhofari Arabs of Southern Oman** | 420 | 0.000 (0.000) | 0.724 (0.022) | ***44*** |
| **Yemenis** | 478 | 0.002 (0.002) | ***0.548 (0.02)*** | ***44*** |
| **Saudi Arabs** | 864 | 0.002 (0.002) | ***0.594 (0.02)*** | ***44*** |
| **UrbanArabs Palestinians** | 81 | 0.025 (0.017) | 0.049 (0.024) |  |
| **Druze Palestinians** | 14 | 0.036 (0.049) | 0.107 (0.083) | ***43*** |
| **Bedouin Palestinians** | 19 | 0.026 (0.037) | 0.132 (0.078) | ***43*** |
| **Palestinians** | 18 | 0.028 (0.039) | 0.00 (0.000) |  |
| **Saudi Arabia Bedouin** | 46 | 0.000 (0.000) | ***0.489 (0.074)*** | ***43*** |
| **Jordan Bedouin** | 23 | 0.065 (0.051) | ***0.391 (0.102)*** | ***43*** |
| **Sudan Beni Amir** | 82 | 0.006 (0.009) | 0.244 (0.047) | ***43*** |
| **Sudan Shaigi** | 9 | 0.000 (0.000) | 0.056 (0.077) |  |
| **Sudan Dounglawi** | 6 | 0.000 (0.000) | 0.00 (0.000) |  |
| **Sudan Jaali** | 88 | 0.006 (0.008) | 0.142 (0.037) | ***41*** |

^a^Number of chromosomes. ^b^Arabs: form Syria, Iraq, Lebanon, and Palestine. N.D. = not determined
